# Isolation and characterization of multidrug resistant *Gallibacterium anatis* biovar* haemolytica* strains from Polish geese and hens

**DOI:** 10.1186/s13567-023-01198-2

**Published:** 2023-08-23

**Authors:** Magdalena Karwańska, Alina Wieliczko, Anders Miki Bojesen, Kasper Rømer Villumsen, Eva Krzyżewska-Dudek, Anna Woźniak-Biel

**Affiliations:** 1 Department of Epizootiology and Clinic of Birds and Exotic Animals, Faculty of Veterinary Medicine, Wrocław University of Environmental and Life Sciences, Wrocław, Poland; 2 Department of Veterinary and Animal Sciences, Faculty of Health and Medical Sciences, University of Copenhagen, Copenhagen, Denmark; 3 Department of Immunology of Infectious Diseases, Hirszfeld Institute of Immunology and Experimental Therapy, Polish Academy of Sciences, Wrocław, Poland

**Keywords:** *Gallibacterium anatis*, poultry, NGS data, antimicrobial resistance, phylogeny, virulence genes

## Abstract

*Gallibacterium anatis* biovar *haemolytica* is a bacterium that is frequently associated with infections of the reproductive tract and respiratory system in poultry. To assess the current prevalence and resistance profile of these bacteria in Poland, we collected and investigated 63 strains of *Gallibacterium* from diseased domestic poultry flocks including geese, laying hens, breeding hens and an ornamental hen. Detailed characterization of the isolates included the analysis of phenotypic antimicrobial resistance profiles and biofilm formation ability. Furthermore, the genetic background of 40 selected isolates regarding the presence of virulence and antimicrobial resistance genes and mobile genetic elements was determined. All investigated isolates were multidrug resistant, most prominently to β-lactams, fluoroquinolones, sulfonamides and macrolides. A total of 48 different resistance profiles were detected. Of all isolates, 50.8% formed a strong biofilm, where strains isolated from geese appeared to be better at biofilm formation than strains isolated from laying and breeding hens. Single-nucleotide polymorphism genotyping revealed that *G. anatis* bv. *haemolytica* strains are restricted in host and geographical distribution, and the geese isolates showed greater phylogenetic similarity. Whole genome sequencing enabled identification of 25 different antimicrobial resistance determinants. The most common resistance genes were *tetB*, *bla*_*ROB-1*_, and *bla*_*TEM-1*_ which may be located on mobile genetic elements. All isolates possessed the toxin gene *gtxA,* and the fimbrial gene *flfA* was identified in 95% of strains. Our results indicated that all *G. anatis* bv. *haemolytica* isolates showed multidrug resistant phenotypes. Strains isolated from geese were characterized by the highest percentage of isolates resistant to selected antimicrobials, probably reflecting host-related adaptations.

**Supplementary Information:**

The online version contains supplementary material available at 10.1186/s13567-023-01198-2.

## Introduction

For years, Poland has had one of the largest poultry productions in the European Union [1]. The basis for the production of poultry meat and eggs in Poland is the intensive rearing of broiler chickens and laying hens [2]. However, Poland is also the largest European geese producer and is second, just behind China, in the global geese production. The production of geese is an important and growing branch of the Polish poultry industry [3, 4]. The most significant commercial goose is the White Kołuda breed, representing approximately 90% of the country’s goose population [4, 5]. The production of broiler and breeding geese is seasonal and the breeding cycle begins early spring and ends in the autumn [3]. In Poland, geese breeding is carried out in semi-closed breeding systems, in which free access to the external environment and water is allowed. Outdoor access increases the risk of disease transmission from wild birds.

The *Gallibacterium* genus was associated with the *Pasteurellaceae* family in 2003 and comprises four species: *Gallibacterium anatis* (*G. anatis*) (divided into two biovars: *haemolytica* and a non-haemolytic variant), *Gallibacterium melopsittaci* sp. nov., *Gallibacterium trehalosifermentans* sp. nov., *Gallibacterium salpingitidis* sp. nov., and three genomospecies [6–8]. All *Gallibacterium* taxa are commonly isolated from a wide spectrum of avian hosts including poultry and wild birds; although isolates have also been reported from farm animals mainly cattle, pigs, and rabbits [7, 9–11]. *Gallibacterium anatis* biovar *haemolytica* (*G. anatis* bv. *haemolytica*) is the most commonly reported species in poultry [12]. It is a Gram-negative, facultative anaerobic, rod-shaped bacterium that forms a wide β-haemolysis zone around the colony on blood agar plates [7, 13]. Although *G. anatis* is part of the indigenous microbiota in the upper respiratory tract and lower reproductive tract of healthy birds, it is an opportunistic pathogen [8, 14]. *G. anatis* has also been associated with a wide variety of pathological manifestations such as peritonitis, salpingitis, oophoritis, hepatitis, and respiratory syndrome [10, 11, 14, 15]. An abundance of these bacteria in the early growth phase of poultry may result in lowered body weight and dysbiosis, and subsequently impact broiler performance, increased mortality rates, and reduced egg production, thus causing severe economic losses in the poultry industries [14, 16]. Mixed infections with viral pathogens causing e.g. infectious bronchitis (IB), infectious laryngotracheitis (ILT), turkey rhinotracheitis (TRT) or bacteria, such as *E. coli*, *Avibacterium paragallinarum*, *Mycoplasma* spp. likely aggravate the disease process and complicates diagnostic procedures [14, 17, 18].

Many known virulence determinants are present in *Gallibacterium* spp. strains, although the mechanism of pathogenesis of these bacteria is still not fully understood [7, 10, 18]. The most important factors for the virulence of *G. anatis* bv. *haemolytica* are toxins and fimbriae. GtxA is an important component of a specific RTX toxin that has haemolytic and cytolytic activity, as well as immunogenic properties [13, 19]. *Gallibacterium* F-17 like fimbriae are involved in the colonization of the upper respiratory tract and play a crucial role in the pathogenesis [20, 21]. Furthermore, *G. anatis* fimbriae were classified into five types Flf, Flf1, Flf2, Flf3 and Flf4, based on the phylogenies of their subunits [22]. Some *G. anatis* strains also have a very strong ability to produce biofilm [23]. The formation of biofilm enables bacteria to survive in unfavorable environmental conditions and hinders the elimination of microorganisms from the environment of animal husbandry, including poultry farms, or production lines. Biofilm formation is also associated with persistent and chronic infections and increased resistance to antimicrobials [7, 10].

Multidrug resistant strains of *G. anatis* are reported with increasing frequency, showing resistance to many antimicrobials [24–26]. According to the microbiological nomenclature, a MDR strain is defined as being non-susceptible (resistant or intermediate susceptible in vitro) to at least one antimicrobial from three or more classes of antibacterial drugs that are used to treat infections caused by the pathogen [27]. Horizontal gene transfer (HGT), mobile genetic elements (plasmids, transposons), and exposure to antibacterial agents may lead to selection of resistance genes and resistance build-up.

Currently, there are no data on the occurrence or the phenotypic and genotypic characteristics of *G. anatis* strains isolated from various species of poultry in Poland. So far, research in this field relating to Polish production, has been carried out by Rzewuska et al. [9], covering the characteristics of *Gallibacterium* spp. strains isolated from peacocks with respiratory symptoms, and by Stępień-Pyśniak et al. [28] who developed the LAMP method for the rapid and effective identification of *Gallibacterium* strains isolated from poultry. Beyond these studies, research on the molecular characteristics of *G. anatis* bv. *haemolytica* strains isolated from domesticated waterfowl are scarce [6, 29]. Moreover, research by Bojesen et al. [30] and Bisgaard et al. [6] indicated the presence of distinct genetic lineages within the former taxon 2 and 3 complex of Bisgaard that are specific for host-bird species of different families e.g., taxon 2 *Pasteurellaceae* is characteristic for geese.

In light of this limited current state of knowledge, the aim of this study was to characterize *Gallibacterium anatis* bv. *haemolytica* strains isolated from laying, breeding, and ornamental hens, and from geese. All the isolates were investigated with respect to antimicrobial resistance (AMR) profiles, biofilm production capacity, and the prevalence of virulence and resistance genes. This study included a comparison of AMR profiles with antimicrobial agents commonly used in poultry production in Poland. Additionally, mobile genetic elements covering antimicrobial resistance were characterized to investigate the possible transfer of resistance genes between poultry strains and the environment of poultry farms. Finally, an additional objective of this study was to investigate the genetic relationship between all isolates and the genetic diversity per host based on genome sequence comparisons.

## Materials and methods

### Bacterial isolates

**Figure 1:**
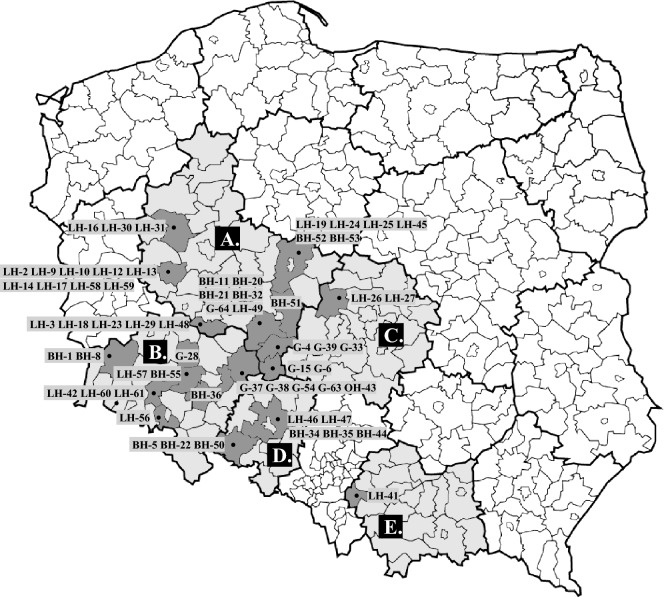
**Geographical distribution of *Gallibacterium anatis* biovar *haemolytica* strains isolated from the five administrative regions (voivodeships).** Strains *G. anatis* bv. *haemolytica* isolated in 2015–2020 from the following voivodships.: **A**—Greater Poland, **B**—Lower Silesian, **C**—Łódź, **D**—Opole, **E**—Lesser Poland.

The study included 63 clinical isolates of *Gallibacterium anatis* biovar *haemolytica* strains isolated from 39 farms belonging to 28 different poultry producers, covering five administrative regions (voivodeships) (Figure 1). These five regions are of key importance for Polish poultry production and have the highest concentration of poultry farms in the country. The bacteria were isolated from internal organs (liver, spleen or heart), trachea, or ovary of laying hens (*n* = 34), breeding hens (*n* = 17), geese (*n* = 11), and an ornamental hen (*n* = 1) between 2015 and 2020 by the AGRO-VET Veterinary Laboratory in Wrocław, Poland. Samples were collected from birds with symptoms of the reproductive or respiratory tract and submitted for microbiological test to the veterinary laboratory. Details of strain isolation source are provided in Table 1.

**Table 1:** ***Gallibacterium anatis*** **bv.** ***haemolytica*** **strains isolated from geese, laying hens, breeding hens and ornamental hen between 2015 and 2020**.

Host	Strain	Isolation year	Isolation source	Age [weeks]	Farm	Poultry house	Localisation (voivodeship)	Production system
Geese (*n* = 11)	G-37*	2019	Organ swab	ND	1		Lower Silesian	Breeding goose
	G-38*	2019	Organs**	17				
	G-54*	2020	Organs**	8–9				
	G-4*	2016	Trachea	5	2		Greater Poland	Broiler goose
	G-39*	2019	Organs**	ND				
	G-6*	2016	Organs**	ND	3		Greater Poland	Broiler goose
	G-15*	2017	Organs**	ND	4		Greater Poland	Breeding goose
	G-28*	2019	Organs**	11–12	5		Lower Silesian	Breeding goose
	G-33*	2019	Organs**	ND	6		Greater Poland	Broiler goose
	G-63*	2019	Ovary	ND	7		Lower Silesian	Broiler goose
	G-64*	2020	Ovary	13	8		Greater Poland	Broiler goose
Laying hens (*n* = 34)	LH-9*	2017	Organs**	27	9 A	K8	Greater Poland	
	LH-10	2017	Organs**	27		K9		
	LH-12*	2017	Organs**/ovary	30		K3		
	LH-13	2017	Organs**/ovary	30		K5		
	LH-14*	2017	Organs**/ovary	30		K6		
	LH-17 *	2018	Trachea	30				
	LH-16	2018	Trachea	8	9 B		Greater Poland	
	LH-30*	2019	Trachea	22		K6		
	LH-31	2019	Trachea	24		K7		
	LH-19*	2018	Trachea/ovary	20	9 C	K7	Greater Poland	
	LH-24	2019	Organs**	37		K5		
	LH-25	2019	Organs**	23		K10		
	LH-45	2020	Trachea	25				
	LH-26	2019	Trachea	10–11	9 D	K12	Łódź	
	LH-27*	2019	Trachea	10–11		K13		
	LH-3*	2016	Trachea	16	9 E		Greater Poland	
	LH-18	2018	Trachea	25		B14		
	LH-23*	2019	Organs**/ovary	40		K.A1		
	LH-29	2019	Trachea	20		K4		
	LH-2*	2016	Trachea	6	9 F		Greater Poland	
	LH-58	2020	Organs**	11		K4		
	LH-59*	2020	Organs**	12		K6		
	LH-60	2020	Ovary	50	9 G	K-52	Lower Silesian	
	LH-61*	2020	Organs**	50		K-52		
	LH-42	2019	Ovary	39	9 H	K20	Lower Silesian	
	LH-40*	2019	Trachea	20	10		ND	
	LH-41*	2019	Trachea	44	11		Lesser Poland	
	LH-48	2020	Organs**	25	12		Greater Poland	
	LH-49	2020	Organs**	21	13		Greater Poland	
	LH-57*	2020	Organs**	44	14		Lower Silesian	
	LH-56	2020	Trachea	42	15	WW-2	Lower Silesian	
	LH-46	2020	Trachea	44	16 A	K5	Opole	
	LH-47*	2020	Trachea	44		K10		
	LH-7	2017	Trachea	42	17		ND	
Breeding hens (*n* = 17)	BH-1*	2015	Trachea	ND	9 I		Lower Silesian	
	BH-8*	2017	Organs**	ND	9 J		Lower Silesian	
	BH-22*	2019	Trachea	44	16 B	K2	Opole	
	BH-34	2019	Trachea	35	16 C	K1	Opole	
	BH-35*	2019	Trachea	35		K2		
	BH-5*	2016	Trachea	45	18		Opole	
	BH-50 *	2020	Trachea	60				
	BH-20 *	2018	Trachea	20	19	K1	Greater Poland	
	BH-21	2018	Trachea	41		K3		
	BH-55	2020	Trachea	30	20		Lower Silesian	
	BH-11	2017	Trachea	10	21		Greater Poland	
	BH-36*	2019	Ovary	32	22		Lower Silesian	
	BH-44*	2020	Ovary	35	23		Opole	
	BH-51*	2020	Organs**	21	24		Greater Poland	
	BH-52	2020	Trachea	42	25		Greater Poland	
	BH-53*	2020	Trachea	42	26		Greater Poland	
	BH-32*	2019	Trachea	29	27		Greater Poland	
Ornamental hen (*n* = 1)	OH-43*	2019	Ovary	ND	28		Lower Silesian	

^*^Strains selected for whole genome sequencing.

^**^Strains isolated from internal organs: liver, spleen or heart.

9A—9 J—poultry farms from the same poultry producer; 16A—16C—poultry farms from the same poultry producer.

All isolates were inoculated on Columbia agar (Oxoid, Hampshire, UK) supplemented with 5% sheep blood and incubated aerobically at 37 °C for 24 h. All 63 isolates were identified by matrix-assisted laser desorption ionization-time of flight mass spectrometry (MALDI-TOF MS) using MALDI Biotyper v.3.1 software (Bruker Daltonics GmbH, Bremen, Germany) and PCR targeting 16S rRNA and 23S rRNA genes as previously described [31, 32]. The reference strain *Gallibacterium anatis* DSM 16844 was used as a positive control in the PCR reaction and MALDI-TOF analysis, and was obtained from the German Collection of Microorganisms and Cell Cultures at the Leibniz Institute. All collected strains were saved for further analysis and stored at −70 °C using BHI broth (Oxoid, Hampshire, UK) supplemented with 40% glycerol.

### Antimicrobial resistance

Antimicrobial susceptibility to selected antimicrobials commonly used in the treatment of avian and poultry-specific bacterial infection but also in human medicine (ENRO – enrofloxacin, GEN—gentamicin, XNL—ceftiofur, NEO—neomycin, ERY—erythromycin, OXY—oxytetracycline, TET—tetracycline, AMOX—amoxicillin, SPE—spectinomycin, SDM—sulfadimethoxine, SXT—trimethoprim/sulfamethoxazole, FFN—florfenicol, STZ—sulfathiazole, PEN—penicillin, STR—streptomycin, NOV—novobiocin, TYLT—tylosin tartrate, CLI—clindamycin) was tested to establish the MIC (Minimum Inhibitory Concentration) by the Sensititre Avian AVIAN1F Vet AST Plate (Thermo Fisher Scientific, Waltham, MA, USA). Additionally, all *G. anatis* bv. *haemolytica* isolates were investigated for sensitivity to antimicrobials dedicated to *Enterobacteriaceae* (SMX—sulfamethoxazole, TMP—trimethoprim, NAL—nalidixic acid, CIP—ciprofloxacin, TET—tetracycline, MERO—meropenem, AZI—azithromycin, FOT—cefotaxime, CHL—chloramphenicol, TGC—tigecycline, TAZ—ceftazidime, COL—colistin, AMP—ampicillin, and GEN—gentamicin) using the Sensititre, EU Surveillance *Salmonella*/*E. coli* EUVSEC AST Plate (Thermo Fisher Scientific, Waltham, MA, USA) allowed broadening of the spectrum of tested antimicrobials. The dilutions of antimicrobials used are presented in a supplementary table (Additional file 1). The broth microdilution method was done according to the manufacturer’s instructions using cation adjusted Mueller Hinton broth with TES (Thermo Fisher Scientific, Waltham, MA, USA). *Escherichia coli* ATCC 25922 was used as the internal control for both tested AVIAN1F Vet AST and EUVSEC AST plates*.* Antimicrobial resistance phenotyping of isolates was performed and interpreted according to the Clinical and Laboratory Standards Institute (CLSI) VET06 ED1:2017 [33]*.* Due to the lack of breakpoints for the genus *Gallibacterium* for some antimicrobials the MIC values were determined based on guidelines for the *Pasteurellaceae* family according to the Clinical Laboratory Standards Institute (CLSI) M100 ED32:2022 and the European Committee on Antimicrobial Susceptibility Testing (EUCAST) version 12.0 [34, 35].

### Biofilm formation

Biofilm formation was examined using the method of O’Toole and Kolter with a few modifications [36]. Strains were pre-cultured overnight in BHI medium (Oxoid, Hampshire, UK) and adjusted to an optical density of 1 McFarland and diluted 1:100 in fresh BHI medium. A volume of 200 µL of each dilution was dispensed into a 96-well microtiter plate in five technical replicates per strain. The plates were incubated at 37 °C for 24 h. Washing and staining were performed as previously described [36]. Optical density was measured at 570 nm (OD570) with a Spark microtiter reader (TECAN, Männedorf, Switzerland). Each strain was tested in two independent biological repetitions. The interpretation of biofilm production was done according to the criteria by Stepanović [37] as follows: OD ≤ ODc non-biofilm producer; ODc < OD ≤ 2ODc weak biofilm producer; 2ODc < OD ≤ 4ODc moderate biofilm producer; 4ODc < OD strong biofilm producer. Here, OD represents the average optical density and the cut-off ODc was defined as three standard deviations above the mean OD of the negative control. Negative controls for the test were uninoculated BHI medium.

### DNA isolation

Bacteria were incubated in BHI broth (Oxoid, Hampshire, UK) at 37 °C, for 24 h. Genomic DNA was extracted using mini-columns from commercially available Genomic Mini Kit (A&A Biotechnology, Gdańsk, Poland) according to the manufacturer’s instructions. The DNA concentration and quality was measured using a NanoDrop spectrophotometer (DeNovix, Wilmington, USA) and stored at −20 °C for further analysis.

### Whole-genome sequencing

Of the 63 strains included in this study, a total of 40 strains were whole-genome sequenced. Of these 40 strains, 34 were sequenced by Macrogen (NovaSeq 6000 (Illumina), 150 bp paired-end sequencing) Seoul, Korea) and six were sequenced at the Department of Veterinary and Animal Sciences, Faculty of Health and Medical Sciences, University of Copenhagen, ((MiSeq (Illumina), 300 bp paired-end sequencing), Copenhagen, Denmark). The sequenced strains include all strains isolated from geese (*n* = 11) and the single isolate obtained from an ornamental hen (*n* = 1). In addition to these, 28 isolates obtained from the laying (*n* = 16) and breeding (*n* = 12) hens were selected to provide the most diverse data possible regarding: the origin of the strain, the time of isolation, the location of the farm and the differences in the antimicrobial resistance profiles, taking into account each geographical location studied. In addition, if possible, strains isolated in different years from the same farm were chosen for whole genome sequencing (WGS). Sequence data of the forty *G. anatis* bv. *haemolytica* strains have been deposited in the NCBI Sequence Read Archive (SRA) under BioProject PRJNA929704, with accession numbers SRX19225671—SRX19225710.

### Bioinformatic analyses

Raw sequence reads were assessed for general quality using FastQC (v0.11.9) [38] and FastQ Screen (v0.11.3) [39], and summarized using MultiQC (v1.12) [40]. The reads were then trimmed for adapter sequences, as well as quality using a sliding window approach with a cut-off at phred score 20 and a window size of 4 using AdapterRemoval (v2.3.2) [41]. For phylogenetic analysis, a core single nucleotide polymorphisms (SNP)-based mapping analysis was performed using Snippy (v4.6) [42], comparing all trimmed reads to the *G. anatis* reference strain UMN179 (Genbank accession no. CP002667) [43], followed by isolation of variant sites using snp-sites (v.2.5.1) [44] and finally the phylogenetic tree was constructed using a generalized-time reversible algorithm in FastTree (v2.1.11) [45] and visualized using iTol (v6.6) [46].

Draft genomes were assembled using MEGAHIT (v1.2.9) [47]. Quality and completeness were assessed by remapping raw reads onto the assembled genomes using bwa (v 0.7.17-r1188) [48] and samtools (v1.15) [49], as well as using QUAST (v5.0.2) [50] and CheckM via kbase.us [51]. AMR and virulence genes were identified using AMRFinderPlus (v3.10.20) [52], mobile genetic elements were identified using MobileElementFinder (v1.0.3, database v1.0.2) [53] and each draft genome was annotated using Prokka (v1.14.6) [54] including the amino acid sequences of *G. anatis* proteins GtxA and FlfA (UniProtKB accessions F4HD31 and L0L6D6, respectively) as trusted proteins during the annotation.

## Results

### Identification

All 63 isolates were identified as *G. anatis* bv. *haemolytica* based on circular, raised greyish, shiny, and semitransparent colony morphology with β-haemolysis; the MALDI-TOF MS technique with a log score value greater than 2.0, and further confirmed by PCR reaction. The general description of 63 strains is summarized in Table 1 and in Figure 1.

### Antimicrobial resistance

**Figure 2:**
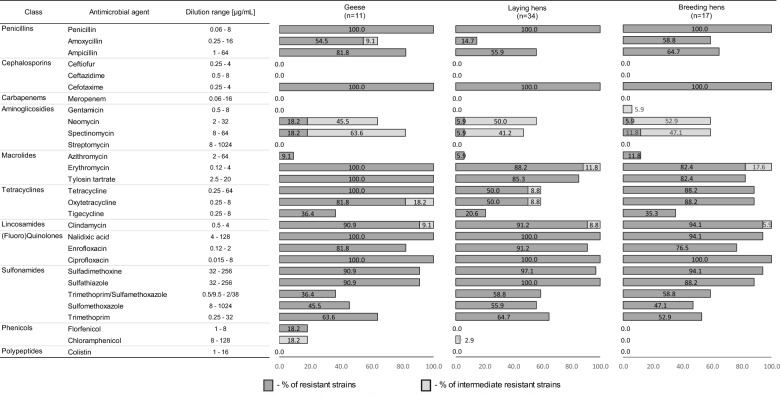
**Resistance [%] of *Gallibacterium anatis* biovar *haemolytica* strains to selected antimicrobial agents.** Figure presenting the differences in resistance profiles among poultry species geese (*n* = 11), laying hens (*n* = 34) and breeding hens (*n* = 17).

All *Gallibacterium anatis* bv. *haemolytica* strains were resistant to penicillin, cefotaxime, and ciprofloxacin (Figure 2). Most of the isolates were resistant to quinolones such as enrofloxacin (98.4%) and nalidixic acid (85.7%), followed by sulfonamides: sulfamethoxazole (95.2%) and sulfadimethoxine (95.2%), as well as to clindamycin (92.1%). High MIC values were also recorded against macrolides where 88.9% of strains were resistant to erythromycin and 87.3% were resistant to tylosin tartrate. A high number of isolates resistant against oxytetracycline (68.2%) and tetracycline (65.1%) were also observed. In contrast, a low percentage of isolated *G. anatis* bv. *haemolytica* strains were resistant to spectinomycin, neomycin, florfenicol—9.5%, 7.9%, and 3.2%, respectively. Intermediate resistance was demonstrated in a minor subset of the isolates, namely towards neomycin (49.2%), spectinomycin (47.6%), erythromycin (11.1%), clindamycin (7.9%), tetracycline (7.9%) and oxytetracycline (4.8%); additionally, three strains (4.8%) were intermediate resistant to chloramphenicol and one each (1.6%) to amoxicillin and gentamicin. Finally, all isolates (100%) were susceptible to the cephalosporins: ceftiofur and ceftazidime, as well as to meropenem, streptomycin, and colistin.

Compared to laying and breeding hens, the frequency of resistance to ampicillin, neomycin, spectinomycin, erythromycin, tylosin tartrate, tetracycline and florfenicol was notably higher in the geese isolates. Notwithstanding, a higher percentage of strains isolated from laying and breeding hens showed resistance to sulfadimethoxine, trimethoprim/sulfamethoxazole, and sulfamethoxazole (Figure 2, Additional file 1).

All 63 *G. anatis* bv. *haemolytica* isolates were multidrug resistant. The *Gallibacterium* isolates used in this study showed 48 different resistance profiles to antibacterial agents; however, no dominant resistance pattern could be identified for this. The most common AMR profile consisted of eight classes of antimicrobial agents: PEN AMP FOT NEO SPE OXY TET ERY TYLT CLI ENRO NAL CIP SDM STZ SXT SMX TMP and represented by four strains, three from laying hens and one from geese (Additional file 2).

The most common AMR profile consisted of eight classes of antimicrobial agents: PEN AMP FOT NEO SPE OXY TET ERY TYLT CLI ENRO NAL CIP SDM STZ SXT SMX TMP and was represented by four strains, three from laying hens and one from geese. Repeating profiles PEN AMOX AMP FOT NEO SPE OXY TET ERY TYLT CLI ENRO NAL CIP SDM STZ SMX and PEN AMP FOT NEO SPE OXY TET TGC ERY TYLT CLI ENRO NAL CIP SDM STZ SXT SMX TMP were also observed among three strains from laying and breeding hens. Furthermore, eight pairs of repeating AMR profiles were identified among sixteen strains that were isolated mainly in birds that came from different farms of the same poultry producer. A unique AMR profile was found only in 37 isolates of *G. anatis* bv. *haemolytica*.

The geese isolates G-37 and G-38 were resistant to the highest number of twenty one different antimicrobial agents belonging to nine different classes; in contrast, strain LH-17, isolated from a laying hen, was resistant to the lowest number of nine antimicrobial agents from six different classes: PEN FOT ERY TYLT CLI ENRO CIP SDM STZ.

### Biofilm formation

All 63 *G. anatis* bv. *haemolytica* isolates were able to form biofilm on polystyrene surfaces (Additional file 2). 50.8% of all investigated isolates formed strong biofilm, including 90.9% of geese strains, whereas that was the case for 47.1% of the breeding hens strains and 41.2% of laying hens isolates. In contrast, only eight strains (12.7%) were characterized by weak biofilm growth, hereof six strains from laying hens (17.6%) and two strains from breeding hens (14.3%). For 41.2% of the strains isolated from laying and breeding hens a moderate biofilm production was recorded. Moderate biofilm production was observed in one goose isolate and the strain isolated from the ornamental hen. The control strain *G. anatis* DSM 16844, as shown in previous studies, formed a strong biofilm [20].

### Phylogenetic tree

**Figure 3:**
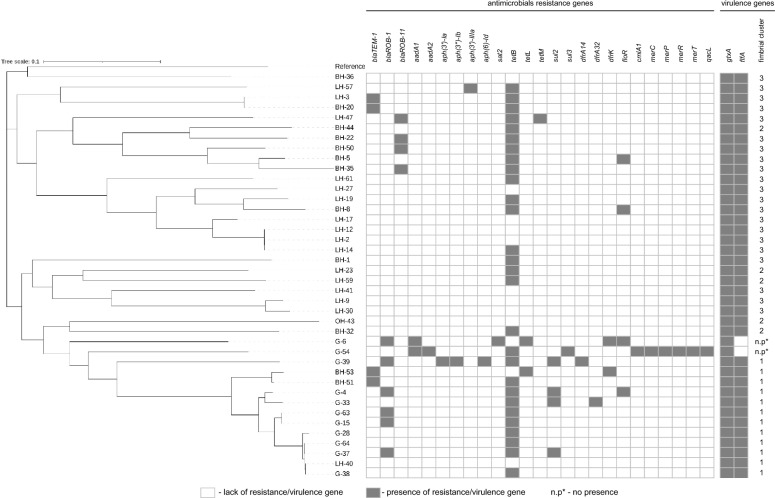
**Phylogeny of *Gallibacterium anatis* biovar *haemolytica* based on single-nucleotide polymorphism (SNP) genotyping**. Presence SNP phylogeny and occurrence of detected antimicrobial resistance genes and virulence genes in the forty sequenced isolates.

A phylogenetic tree was constructed from the core genome SNP distances based on the 40 sequenced poultry isolates and the reference strain *G. anatis* UMN179 (Figure 3). The phylogenetic tree comprised seven clades and three distinct subtrees, which generally appeared associated with the host type from which they were isolated. The resulting tree includes strains isolated from geese and strains isolated from chickens: BH-51, BH-53 and LH-40 in one cluster. Strains G-28, G-38, G-37, LH-40 and G-64, located on a separate branch, show the greatest similarity. A separate clade consisted of the following strains: BH-32 showing resistance to the lowest number of antimicrobials tested among all breeding hen isolates, and the strain obtained from ornamental hen OH-43. The sequence of the reference strain (UMN179) was a separate clade with the BH-36 strain. Isolates LH-57, BH-20 and LH-3 of different origins belonged to two separate lineages, one including strains LH-57, and another lineage based on strains BH-20 and LH-3. The strains LH-61, LH-17, LH-12, LH-14, LH-2, LH-27, BH-8, LH-19 of mixed origin clustered together, they came from different farms belonging to one poultry parent company in Poland. The six strains, LH-47, BH-50, BH-35, BH-5, BH-22, BH-44, made up yet another cluster of highly similar strains which were isolated exclusively from within the Opole voivodeship.

### Genome assembly

Following initial quality control and subsequent trimming of sequencing reads, draft assemblies were made for each isolate. Across all 40 included strains, the average draft genome size was 2.51 Mb in length, with a 40% GC-content which is in agreement with that reported by Johnson et al. [43]. Furthermore, N50 values ranged from 45,414–112089, with a mean of 73 773. A mean 99.89% of trimmed reads were successfully mapped onto to their respective draft assemblies, and all 40 draft genomes had an estimated 100% genome completeness. Only LH-14 was found to have any estimated genome contamination (1.13%), as estimated by CheckM [58]. The detailed characteristic of the 40 strains selected for the WGS studies is presented in Table 1. See Additional files 2 and 3 for full details.

### Resistance genes

The presence of antimicrobial resistance genes in the 40 genome sequences was investigated by AMRFinder Plus (Figure 3). A total of 25 different resistance genes were identified. The resistance genotypes included tetracycline, sulfonamides and trimethoprim, penicillins, aminoglycosides and florfenicol. The most common resistance gene was *tetB*, which occurred in twenty eight (70%) of the strains. In addition, two strains carried the *tetL* gene and encoded both the *tetB* and *tetM* resistance genes. The presence of tetracycline resistance genes correlated with phenotypic resistance, of the 30 strains possessing the tetracycline resistance gene, only one was phenotypically sensitive to this antibiotic. The prevalence of penicillin resistance genes was as follows: the *bla*_*TEM-1*_ gene was found in four (10%) of the total strains and *bla*_*ROB-1*_ in six (15%) strains, of only goose origin. The *bla*_*ROB-11*_ gene was detected in four laying and breeding hen strains isolated from a single Polish region—Opole voivodeship. Furthermore, the florfenicol resistance gene *floR* was observed in four strains and antimicrobial susceptibility tests confirmed resistance to florfenicol. Among the *G. anatis* bv. *haemolytica* isolates obtained from geese, the presence of sulfonamides resistance genes *sul2* and *sul3* was observed in four and one strains, respectively. The goose isolates appeared to harbor a higher number of resistance genes responsible mainly for resistance to trimethoprim and aminoglycosides: *dfrK* (*n* = 2), a*adA1* (*n* = 2) and *aadA2*, *aph(3)-la*, *aph(3)-lb*, *aph(6)-ld*, *sat2*, *dfrA14*, *dfrA32* (*n* = 1). Isolate G-54 carried *merC*, *merP*, *merR*, *merT* genes belonging to the mercury resistance operon, *qacL*—quaternary ammonium compound efflux transporter and *cmlA1* gene that confers resistance to chloramphenicol.

### Mobile genetic elements

In 36 of the 40 strains, a total of 61 insertion sequences (IS) were identified, belonging to three different IS families: 1016, 10 and 5. These sequences included eight distinct types of insertion elements, of which the strains isolated from laying and breeding hens carried the IS elements such as ISGasp1, ISNesp1, ISVsa5, ISApl1, ISGan1, while for the geese isolates ISGan1, ISKpn13 and IS26 were common. Two strains, LH-3 and BH-20, encoded a Tn2 transposon containing the *bla*_*TEM-1*_ gene, which determines resistance to antibiotics from the B-lactamase class. Additionally, in BH-22, BH-35 and BH-50 strains isolated from breeding hens the gene *bla*_*ROB-1*_ was identified in a cn_3526_ISApl1 composite transposon. Transposon cn_6122_ISVsa5, which was detected in strain LH-61 contained the *tetB* gene. Sequence data from one strain G-54 isolated from geese contained the ColpVC plasmid (Additional file 4).

### Virulence genes

The sequences from the *G. anatis* bv. *haemolytica* strains were screened for the best-known virulence genes *gtxA* and *flfA*. Our analysis indicated that all isolates possessed the *gtxA* gene corresponding to the presence of a haemolytic RTX toxin. One to three copies of the virulence gene *flfA* was identified in 38 strains (95%) (Figure 3). The *flfA* gene was not detected in 2 isolates of geese G-6 and G-54. All remaining geese strains and isolates BH-51, BH-53, and LH-40 had one copy of the *flfA* gene with the same length of 528 bp. The strains: BH-32, BH-44, LH-23, LH-59, and OH-43 had two copies, while the rest of the strains isolated from laying and breeding hens had three copies of the *flfA* gene responsible for the fimbrial biosynthesis.

## Discussion

Worldwide the increase of antimicrobial resistance makes the selection of effective antimicrobial therapy for animals increasingly difficult, pointing to the importance to monitor phenotypic and genotypic susceptibility profiles. One of the objectives of our study was to determine and compare the resistance of *G. anatis* bv. *haemolytica* isolates to commonly used antimicrobial agents. Our study showed that all strains tested from both laying and breeding hens as well as from geese, were multidrug resistant. Similarly, high percentages of isolates resistant to multiple classes of antimicrobials among field strains were observed in previous studies [24, 26, 55]. Strains isolated in Poland had decreased susceptibility to: penicillins, tetracyclines, macrolides, sulfonamides, quinolones, which are all antimicrobials that have been used for years in the treatment of human and animal infections. Importantly, resistance to antimicrobial agents of these classes is widespread among the genus *Gallibacterium* and has been described in earlier works from Denmark, USA, Morocco, Egypt, Austria, Belgium and Iran [8, 11, 24–26, 55, 56].

The *Gallibacterium* strains investigated in the current study had diverse AMR profiles. This could likely be due to the varied origins of the strains in the collection, but even strains isolated from a single farm differed. Similar findings of clonal diversity were reported by Hess et. al. [26], who showed that different clones with individual resistance profiles could even be isolated within isolates from different organs of the same individual. In support of that, Bojesen et al. [55], as well as Lozica et al. [15], indicated that *G. anatis* strains may vary phenotypically and genotypically within a single flock of birds.

Furthermore, our research indicated that the revealed resistance profiles of *G. anatis* bv. *haemolytica* were correlated with regimens of antimicrobial use in poultry. In Poland, diseased poultry flocks are treated mainly with polymyxins, penicillins, tetracyclines and fluoroquinolones [57, 58]. To our knowledge, in the case of laying and breeding hens, these are mainly colistin, tiamulin, lincospectin, tylosin, doxycycline, tetracycline, and enrofloxacin, and the treatment of birds is performed according to antibiogram results and in compliance with the withdrawal period, especially in laying hens. In the case of the *G. anatis* bv. *haemolytica* isolates we studied, we did not have access to information or protocols on the current treatment of birds or in the past; therefore, a detailed analysis of possible causes of antimicrobial resistance of strains was not possible.

In our study, strains isolated from laying hens formed the largest and most diverse group of the tested isolates of *G. anatis* bv. *haemolytica*. The difficulty in interpreting the results is that the strains were isolated from farms with different levels of biosecurity measures and management practices. Among laying hens, as many as 25 strains came from one poultry producer, among these isolates six pairs of repeating AMR profiles were observed. These results may suggest that isolates obtained from birds that belonged to the same producer, but were found on different farms, may be related. The probable reason for this may be the movement of farm workers and the low level of biosecurity, which is conducive to the spread of bacterial pathogens or the use of similar treatment protocols within the flocks of the same poultry producer. Taking this into account, it seems that the basic pillar in preventing the spread of pathogens is the biosecurity of farms, high standards of flock management, ensuring the right environment, and caring for the welfare of birds are important tools for the prevention, spread, and control of bacterial infections, including those caused by bacteria such as *Gallibacterium* [15].

Compared to the strains of *G. anatis* bv. *haemolytica* isolated from laying and breeding hens, the geese isolates showed a higher percentage of resistant strains to amoxicillin, ampicillin, erythromycin, tylosin tartrate, tetracycline, and florfenicol; which may be due to the fact that those antimicrobials are widely used in the treatment of bacterial infection in geese [59–61]. Geese housing systems are semi-open, where both geese broilers and breeding geese have access to free range [3]. These birds, therefore, have access to the outdoors and the wild animals that inhabit it. This increases the possibility of transmission of potential microbial pathogens. In combination with the above-mentioned treatment strategies, this increased access to surrounding environment and wildlife could result in an increased transmission and sustained presence of resistance genes. Moreover, our study indicated that strains from geese possessed a higher number of as many as twenty different resistance genes while isolates from laying and breeding hens possessed only eight.

The strain isolated from ornamental hen was resistant towards penicillins, macrolides, quinolones, cefotaxime, and clindamycin, and was sensitive to all drugs of aminoglycosides, tetracyclines, and sulfonamides classes. Although this strain was isolated in an organic farm with no use of any antimicrobial agents, it had the MDR phenotype. The obtained results indicate that the phenotypic resistance of the strains is not exclusively conditioned by the current pressure of the antimicrobials used but may result from the possibility of contact of birds kept in the free-range system with the feces of wild birds. Interestingly, no antimicrobial resistance genes were detected in this isolate, which could be related to the limitation of the bioinformatic tools and approach that we have used.

The phenotypic resistance of *Gallibacterium anatis* bv. *haemolytica* strains were combined with results obtained by sequence-based methods that allowed the identification of antimicrobial resistance genes and the detection of mobile genetic elements (MGE). Our studies led to a better understanding of the mechanisms determining the resistance of *G. anatis* bv. *haemolytica* isolates. We have identified 25 different resistance genes, some of which have already been described in strains isolated from calves [11] and poultry [55, 62]. In addition, the high frequency and diversity of resistance genes in the *G. anatis* bv. *haemolytica* isolates we analyzed suggest that this species can acquire resistance genes easily compared to other *Pasteurellaceae* species [11, 63].

Our study showed that among the antimicrobials tested, the highest percentage of strains wereresistant to quinolones, from 85.7% to 100%. The increase in resistance to this class of antimicrobials was also indicated by two recent studies [26, 56], which showed a significantly higher number of resistant strains compared to earlier studies conducted between 2013 and 2018 [8, 24, 64]. Our study did not demonstrate quinolone resistance proteins that determine resistance to this class of drugs, suggesting that chromosomal mutations in the genes *gyrA*, *gyrB*, and *parC* may be responsible for quinolone resistance [11, 65].

Our research confirmed resistance of *G. anatis* bv. *haemolytica* strains to antimicrobials from the macrolide class, but no resistance genes that determine the above phenotype were detected, while in the study by Van Driessche et al. [11], the presence of the *ermB* gene was confirmed in all investigated isolates, and *mphE* and *mrsE* genes were detected less frequently only in 10% isolates.

The most commonly described resistance, both among *G. anatis* strains isolated from poultry and calves, was tetracycline resistance [11, 55, 56], for which, as in previous studies, the main determinant of resistance to this class of antimicrobials was the *tetB* gene.

In our study, there was a high percentage of resistance to antibiotics from the beta-lactam classes, which was also previously demonstrated by Nassik and Yen [25, 66]. On the other hand, Hess et al. [26] reported a much lower percentage of resistance to ampicillin (19.9%) and to amoxicillin (28.6%) among *G. anatis* strains, which may be due to the use of different treatment regimens for bacterial infections. In our results, resistance to the above antibiotics correlated with the frequency of genes encoding beta-lactamases: *bla*_*TEM-1*_ and *bla*_*ROB-1*_ and *bla*_*ROB-11*_, although resistance to penicillins can also be conditioned by the activity of other beta-lactamases such as bla_OXA-10_ and bla_PSE-1_ [67].

Earlier reports described that, the majority of tested *G. anatis* isolates were resistant to sulfamethoxazole [8, 64], similarly, among isolates used in this study, as many as 95.2% of strains were resistant to this antimicrobial. In addition, strains isolated from geese confirmed the presence of *sul2*, *sul3* and trimethoprim resistance determinant genes *dfrA14*, *dfrK* and *dfrA32*, of which the *sul3*, *dfrK* and *dfrA32* genes were described for the first time in *Gallibacterium* species.

Resistance to florfenicol was detected only among isolates from geese (18.2%), while four strains—two isolates from geese and two from laying hens carried the *floR* resistance gene. Only strain G-4 had a florfenicol resistance phenotype with the *floR* gene. This may be due to the fact that this antimicrobial is mainly used to treat respiratory infections in these birds. Previous work from Morocco and Egypt [8, 52, 62] did not show resistance to florfenicol among *Gallibacterium* strains.

It is worth noting that most studies showed a high percentage of *G. anatis* strains sensitive to aminoglycosides and cephalosporins [8, 23, 24, 26]. Similar observations were noted in our study, the strains were sensitive to antibiotics from these classes. However, all of the *G. anatis* bv. *haemolytica* strains we tested were resistant to cefotaxime. This may be due to the limitation of the method for determining MIC values. The MIC value determined for this antibiotic according to the EUCAST 2022 guidelines [35] for *Pasteurella multocida* species, indicated that strains were considered resistant already at the level > 0.03 mg/L.

In the present study, differences between genotypic and phenotypic resistance to selected antimicrobial agents were observed. Although genotypic assays have been and are widely used to detect AMR genes, there are fundamental differences between a database-screen detection of a gene or a point mutation that could theoretically impart resistance, and actual, functional phenotypic resistance. Thus, the limited overall sensitivity of genotypical screens still makes it difficult to identify some resistance genes and use them as reference AST methods. In line with these arguments, the genotype–phenotype mismatch could have occurred because inducible genes that underwent mutations, insertions, or deletions could be silenced. Although we can often demonstrate that at least some of the mutations and genes identified in vitro are present in resistant clinical isolates, it is difficult to determine whether these are the only or indeed the most relevant genetic factors associated with resistance in these strains [68].

Recent studies showed that genes encoding resistance mechanisms were found on plasmids and other mobile genetic elements, indicating that horizontal gene transfer can occur not only between strains of the same species, but also between different bacterial species, e.g.: including widely distributed strains o*f E. coli* [69]. In addition, the presence and use of antimicrobials is a key determinant of the persistence of resistant subpopulations, and in the poultry farm environment, this will create a selective pressure for the emergence and persistence of antimicrobial resistant strains, including *G. anatis* strains.

In our study, resistance genes of the tested *G. anatis* bv. *haemolytica* isolates were detected at different locations in the genome and were rarely included in MGEs, as previously shown by Johnson et al. [23]. An association of antimicrobial resistance genes with transposons was observed for beta-lactam resistance genes: *bla*_*ROB-11*_, included in the composite transposons detected in isolates BH-22, BH-35 and BH-50 isolated from the Opole voivodeship, and the *bla*_*TEM-1*_ gene, located on the Tn2 transposon, identified in strains BH-20 and LH-3 that shared phylogenetic similarities and clustered together on a separate branch of the phylogenetic tree. Previous studies have shown the presence of *bla*_*TEM*-1_ gene sequences in Tn2 transposons in commensal ampicillin-resistant *E. coli* strains [70]. In addition, one strain of LH-61 had the *tetB* gene localized to a composite transposon, with the localization of this gene to a transposon previously described in *Salmonella* [71]. Horizontal transfer by plasmids plays an important role in the spreading of antimicrobial resistance genes. In the sequence data from goose isolate G-54 from the present study, we were able to identify a ColpVC plasmid previously identified in various *Salmonella* serovars [72].

*Gallibacterium* isolates were characterized by varying biofilm production properties [23]. Our study showed that all isolates had the ability to form biofilm, and produced it with high, medium or low intensity. No phylogenetic correlation was observed between strongly and weakly biofilm-forming strains as in Johnson’s study [23]. Our research has shown that the strains isolated from geese are characterized by a higher biofilm formation capacity. Perhaps this is due to the more frequent use of antimicrobials in these birds, whereby the bacteria respond with a strong biofilm growth. Increased biofilm production may also be of key importance for *Gallibacterium* survival in adverse environmental conditions. However, confirmation of these results requires further research with the use of a larger study population.

Detailed studies of genetic content also provide valuable information on Polish isolates of *G. anatis* bv. *haemolytica*. Based on core genome SNP differences among the 40 isolates, the strains grouped into seven clades. Isolates from geese clustered into one clade, which may indicate a common origin of the strains. All of the geese strains used in this study came from a single native geese breed—the White Kołudzka geese, from which up to 98% of the geese population in Poland currently derives its genotype. Further research is needed to determine if the similarity between the geese strains can indicate vertical transmission of *Gallibacterium*, which has already been indicated in earlier studies [73]. In addition, the geese farms from which the strains we studied originated from a single geographic location with a high density of farms and thereby an increased risk of transmission between farms might be possible. The similarity of the strains may suggest correlations between genetic relatedness and spatial distribution among geese strains. A separate phylogenetic group was also formed by strains isolated exclusively from the Opole voivodeship, which indicate a possible correlation between genetic relatedness and spatial distribution among Polish isolates of *G. anatis* bv. *haemolytica*. At the same time, however, within a single poultry producer, or even the same farm, *G. anatis* bv. *haemolytica* isolates from this study were in different clusters and different distantly related branches of the phylogenetic tree, e.g.: strains LH-3 and LH-23, or strains LH-9, LH-12, LH-17. This may indicate that they do not come from a single introduction or epidemic, and there is a large reservoir of circulating *G. anatis* bv. *haemolytica* strains in the poultry farm environment [55, 56].

The NGS sequencing method also enabled the identification of virulence genes: a toxin (*gtxA*) and fimbriae (*flfA*). As in previous studies [56, 62], all strains possessed the gene *gtxA*, which is a RTX toxin responsible for leukotoxic and haemolytic activity in *G. anatis* bv. *haemolytica* [19]. The presence of the toxin is responsible for the host's ability to adhere and produce pro-inflammatory cytokines, causing immunopathological damage to cells. The toxin *gtxA* is secreted by the type I secretory system, and plays a role in apoptosis of host cells infected by *G. anatis* [74].

In our study, a high percentage of strains (95%) encoded the *flfA* genes. From previous reports by Algammal et al. and Allahghadry et al. this gene was detected in 38.3% and 50%, respectively, while in other studies no *flfA* gene was detected [56, 62, 75]. Such a high percentage of strains with the *flfA* gene may be explained due to the fact that all of the isolates in the present study came from clinical cases, while in previous studies *Gallibacterium* isolates were collected from a large number of samples, randomly collected among poultry flocks. The *flfA* gene is an important virulence factor in the pathogenesis of *G. anatis*, plays a role in tissue tropism, and provides an adhesive capacity that enables bacteria to colonize the upper reproductive tract and respiratory system [21].

As in the investigation by Kudirkienė et al. [22], one to three different copies of fimbriae were identified in a single *Gallibacterium* genome. In our study, strains isolated from geese only had a single *flfA* gene copy, while laying and breeding hens had two or three copies, these results may indicate that they also belonged to different fimbriae clusters.

In conclusion, we have performed a detailed study of *G. anatis* bv. *haemolytica* strains isolated from laying hens and breeding hens and, for the first time in Poland, from geese. Our research showed that all tested *G. anatis* bv. *haemolytica* strains were multidrug resistant and had diverse AMR profiles. Additionally, the geese strains showed greater phylogenetic similarity to each other and were characterized by the highest percentage of antimicrobial resistance genes. This may indicate different adaptation strategies for *Gallibacterium* strains depending on the host species, since growth conditions can significantly affect the metabolism, expression of virulence and resistance factors, and adaptation to the environment of bacterial pathogens. Our results clearly indicate that current poultry treatment regimens and use of antibiotics may lead to an increased frequency of antimicrobial resistance in bacteria. In addition, the variability of *Gallibacterium* strains isolated in small geographic areas and even in single farm systems may indicate that existing biosecurity measures need to be further improved. The use of antimicrobial agents is considered the most important factor in the selection of resistant bacteria, including *Gallibacterium* strains, therefore microbiological testing and prudent use of antimicrobials is extremely important.

### Supplementary Information

**Additional file 1: Antimicrobial dilution range of *G. anatis *bv. *haemolytica *(*n *= 63) according to Minimal Inhibitory Concentration (MIC) value. **Breakpoints were adopted from CLSI ver. VET06 [A], CLSI M100:2022 [B] and EUCAST ver. 12.0 [C]. Vertical lines indicate breakpoints, grey square—resistant; light grey square – intermediate resistant; white square—susceptible; X – means that the dilution range marked in EUVSEC/AVIAN plate was not examined for an antimicrobial; *- MIC value greater than maximum tested. A different range of dilutions for a tylosin tartrate (2,5–20 μg/mL, a value ≥20 μg/mL was taken as the breakpoint) and for b trimethoprim/sulfamethoxazole (0.5/9.5–2/38 μg/mL).

**Additional file 2: Overview of results MALDI-TOF MS, AMR profiles, resistance and virulence genes, and biofilm production of all** ***G. anatis*** **bv.** ***haemolytica*** **isolates (*n*** **= 63).**

**Additional file 3: Assembly characterization of forty sequenced *G. anatis *bv. *haemolytica *isolates.**

**Additional file 4: Overview of detected mobile genetic elements of forty sequenced *G. anatis *bv. *haemolytica *isolates.**

## Data Availability

Sequence datasets generated during this study are available through NCBI’s BioProject database under accession number PRJNA929704. The original contributions presented in the study are included in the article and its additional files, further inquiries can be directed to the corresponding authors.
