# Supplementary material for: Isolation and characterization of multidrug resistant Gallibacterium anatis biovar haemolytica strains from Polish geese and hens
Source: Vet Res. 2023 Aug 23;54:67. doi: 10.1186/s13567-023-01198-2 (PMC10463661; doi:10.1186/s13567-023-01198-2)
Supplement: Supplementary file 1 — Additional file 1: Antimicrobial dilution range of G. anatis bv. haemolytica (n = 63) according to Minimal Inhibitory Concentration (MIC) value. Breakpoints were adopted from CLSI ver. VET06 [A], CLSI M100:2022 [B] and EUCAST ver. 12.0 [C]. Vertical lines indicate breakpoints, grey square—resistant; light grey square – intermediate resistant; white square—susceptible; X – means that the dilution range marked in EUVSEC/AVIAN plate was not examined for an antimicrobial; *- MIC value greater than maximum tested. A different range of dilutions for a tylosin tartrate (2,5–20 μg/mL, a value ≥20 μg/mL was taken as the breakpoint) and for b trimethoprim/sulfamethoxazole (0.5/9.5–2/38 μg/mL). [file 13567_2023_1198_MOESM1_ESM.docx]

Additionally file 1. Antimicrobial dilution range of *G. anatis* bv. *haemolytica* (n=63) according to Minimal Inhibitory Concentration (MIC) value.

| Class | Antimicrobial agent | Antimicrobial dilution range (µg/mL) | | | | | | | | | | | | | | | | | Data source |
| --- | --- | --- | --- | --- | --- | --- | --- | --- | --- | --- | --- | --- | --- | --- | --- | --- | --- | --- | --- |
|  |  | 0.015 | 0.03 | 0.06 | 0.12 | 0.25 | 0.5 | 1 | 2 | 4 | 8 | 16 | 32 | 64 | 128 | 256 | 512 | 1024 |  |
| Penicillins | Penicillin | x | x | **0** | **0** | **0** | **0** | **1** | **8** | **12** | **12** | **(30*)** x | x | x | x | x | x | x | [A] |
|  | Amoxicillin | x | x | x | x | **2** | **8** | **11** | **18** | **2** | **1** | **1** | **(20*)** x | x | x | x | x | x | [A] |
|  | Ampicillin | x | x | x | x | x | x | **23** | **15** | **5** | **0** | **0** | **1** | **2** | **(17*)** x | x | x | x | [C] |
| Cephalosporin | Ceftiofur | x | x | x | x | **47** | **12** | **2** | **2** | **0** | x | x | x | x | x | x | x | x | [A] |
|  | Ceftazidime | x | x | x | x | x | **63** | **0** | **0** | **0** | **0** | x | x | x | x | x | x | x | [B] |
|  | Cefotaxime | x | x | x | x | **63** | **0** | **0** | **0** | **0** | x | x | x | x | x | x | x | x | [C] |
| Carbapenem | Meropenem | x | **32** | **13** | **13** | **5** | **0** | **0** | **0** | **0** | **0** | **0** | x | x | x | x | x | x | [B] |
| Aminoglycosides | Gentamicin | x | x | x | x | x | **4** | **12** | **29** | **17** | **1** | **0** | **0** | x | x | x | x | x | [A] |
|  | Neomycin | x | x | x | x | x | x | x | **0** | **9** | **18** | **31** | **2** | **(3*)** x | x | x | x | x | [A] |
|  | Spectinomycin | x | x | x | x | x | x | x | x | x | **11** | **16** | **30** | **5** | **(1*)** x | x | x | x | [A] |
|  | Streptomycin | x | x | x | x | x | x | x | x | x | **61** | **1** | **1** | **0** | **0** | **0** | **0** | **0** | [A] |
| Macrolide | Azithromycin | x | x | x | x | x | x | x | **34** | **10** | **14** | **4** | **0** | **1** | x | x | x | x | [B] |
|  | Erythromycin | x | x | x | **0** | **0** | **0** | **1** | **0** | **6** | **(56*)** x | x | x | x | x | x | x | x | [A] |
|  | Tylosin tartrate ^a^ | x | x | x | x | x | x | x | **1** | **2** | **5** | **25** | **(30*)** x | x | x | x | x | x | [A] |
| Tetracyclines | Tetracycline | x | x | x | x | **0** | **6** | **11** | **3** | **2** | **2** | **12** | **19** | **7** | **(1*)** x | x | x | x | [A] |
|  | Oxytetracycline | x | x | x | x | **0** | **10** | **7** | **2** | **1** | **4** | **(39*)** x | x | x | x | x | x | x | [A] |
|  | Tigecycline | x | x | x | x | **26** | **20** | **13** | **4** | **0** | **0** | x | x | x | x | x | x | x | [B] |
| Lincosamides | Clindamycin | x | x | x | x | x | **0** | **1** | **4** | **9** | **(49*)** x | x | x | x | x | x | x | x | [A] |
| (Fluoro)Quinolone | Enrofloxacin | x | x | x | **1** | **0** | **0** | **0** | **2** | **(60*)** x | x | x | x | x | x | x | x | x | [A] |
|  | Nalidixic acid | x | x | x | x | x | x | x | x | **3** | **4** | **2** | **0** | **12** | **5** | **(37*)** x | x | x | [C] |
|  | Ciprofloxacin | **0** | **0** | **0** | **0** | **0** | **0** | **1** | **1** | **3** | **9** | **x (49*)** | x | x | x | x | x | x | [C] |
| Sulphonamide | Sulfadimethoxine | x | x | x | x | x | x | x | x | x | x | x | **0** | **3** | **1** | **3** | **(56*)** x | x | [A] |
|  | Sulfathiazole | x | x | x | x | x | x | x | x | x | x | x | **0** | **3** | **14** | **16** | **(30*)** x | x | [A] |
|  | Trimethoprim/Sulfamethoxazole ^b^ | x | x | x | x | x | **13** | **6** | **10** | **(34*)** x | x | x | x | x | x | x | x | x | [A] |
|  | Sulfamethoxazole | x | x | x | x | x | x | x | x | x | **0** | **3** | **6** | **5** | **12** | **5** | **22** | **8 (2*)** | [B] |
|  | Trimethoprim | x | x | x | x | **11** | **2** | **3** | **1** | **8** | **4** | **7** | **8** | **(19*)** x | x | x | x | x | [B] |
| Chloramphenicol | Florfenicol | x | x | x | x | x | x | **46** | **15** | **0** | **1** | **(1*)** x | x | x | x | x | x | x | [A] |
|  | Chloramphenicol | x | x | x | x | x | x | x | x | x | **60** | **3** | **0** | **0** | **0** | x | x | x | [B] |
| Polypeptide | Colistin | x | x | x | x | x | x | **63** | **0** | **0** | **0** | **0** | x | x | x | x | x | x | [C] |

Breakpoints were adopted from CLSI ver. VET06 [A], CLSI M100:2022 [B] and EUCAST ver. 12.0 [C]. Vertical lines indicate breakpoints, grey square – resistant; light grey square – intermediate resistant; white square – susceptible; X – means that the dilution range marked in EUVSEC/AVIAN plate was not examined for an antimicrobial; *- MIC value greater than maximum tested. A different range of dilutions for ^a^ tylosin tartrate (2.5 – 20µg/mL, a value ≥20µg/mL was taken as the breakpoint) and for ^b^ trimethoprim/sulfamethoxazole (0.5/9.5 – 2/38µg/mL).
