# Supplementary material for: Isolation and characterization of multidrug resistant Gallibacterium anatis biovar haemolytica strains from Polish geese and hens
Source: Vet Res. 2023 Aug 23;54:67. doi: 10.1186/s13567-023-01198-2 (PMC10463661; doi:10.1186/s13567-023-01198-2)
Supplement: Supplementary file 2 — Additional file 2: Overview of results MALDI-TOF MS, AMR profiles, resistance and virulence genes, and biofilm production of all G. anatis bv. haemolytica isolates (n = 63). [file 13567_2023_1198_MOESM2_ESM.docx]

| Additional file 2. Overview of results MALDI-TOF MS, AMR profiles, resistance and virulence genes, and biofilm production of all *Gallibacterium anatis* biovar *haemolytica* isolates (*n* = 63). | | | | | | | | | |
| --- | --- | --- | --- | --- | --- | --- | --- | --- | --- |
| Host | Strain | Isolation year | MALDI-TOF MS Score Value | MALDI-TOF MS Matched Pattern | Antimicrobial resistance profiles (AMR) | Resistance genes | Virulence genes | Biofilm formation |  |
| Geese | G-37 * | 2019 | 2.162 | G. anatis GD45 GDD | PEN AMOX AMP FOT NEO SPE OXY TET TGC ERY TYLT CLI ENRO NAL CIP SDM STZ SXT SMX TMP FFN | *blaROB-1, sul2, tetB* | *gtxA, flfA* | S |  |
| (*n* = 11) | G-38 * | 2019 | 2.031 | G. anatis DSM16844T | PEN AMOX AMP FOT NEO SPE OXY TET TGC ERY TYLT CLI ENRO NAL CIP SDM STZ SXT SMX TMP CHL | *tetB* | *gtxA, flfA* | S |  |
|  | G-54 * | 2020 | 2.291 | G. anatis GD45 GDD | PEN AMOX AMP FOT SPE OXY TET ERY TYLT CLI ENRO NAL CIP SDM STZ SMX TMP ^A^ | *aadA1, aadA2, cmlA1, merC, merP, merR, merT, qacL, sul3, tetB* | *gtxA* | S |  |
|  | G-4 * | 2016 | 2.322 | G. anatis DSM16844T | PEN AMOX AMP FOT OXY TET ERY TYLT CLI ENRO NAL CIP SDM STZ TMP FFN | *floR, blaROB-1, sul2, tetB* | *gtxA, flfA* | M |  |
|  | G-39 * | 2019 | 2.257 | G. anatis GD45 GDD | PEN AMP FOT NEO SPE OXY TET TGC ERY TYLT CLI ENRO NAL CIP SDM STZ SMX TMP ^B^ | *aph(3)-la, aph(3)-lb, aph(6)-ld, dfrA14, blaROB-1, sul2, tetB* | *gtxA, flfA* | S |  |
|  | G-6 * | 2016 | 2.128 | G. anatis GD45 GDD | PEN AMOX AMP FOT SPE OXY TET ERY TYLT CLI ENRO CIP SDM STZ SXT TMP CHL | *aadA1, dfrK, floR, ROB-1, sat2, tetL* | *gtxA* | S |  |
|  | G-15 * | 2017 | 2.276 | G. anatis DSM16844T | PEN AMOX FOT OXY TET ERY TYLT CLI ENRO CIP SDM STZ | *blaROB-1, tetB* | *gtxA, flfA* | S |  |
|  | G-28 * | 2019 | 2.261 | G. anatis GD45 GDD | PEN AMP FOT NEO SPE OXY TET TGC AZI ERY TYLT CLI ENRO NAL CIP SDM STZ | *tetB* | *gtxA, flfA* | S |  |
|  | G-33 * | 2019 | 2.155 | G. anatis GD45 GDD | PEN AMP FOT NEO SPE OXY TET ERY TYLT CLI ENRO NAL CIP SDM STZ SXT SMX TMP ^C^ | *dfrA32, sul2, tetB* | *gtxA, flfA* | S |  |
|  | G-63 * | 2019 | 2.348 | G. anatis GD45 GDD | PEN AMOX AMP FOT NEO SPE OXY TET ERY TYLT CLI ENRO NAL CIP SDM STZ | *blaROB-1, tetB* | *gtxA, flfA* | S |  |
|  | G-64 * | 2020 | 2.291 | G. anatis GD45 GDD | PEN FOT NEO SPE OXY TET ERY TYLT CLI ENRO NAL CIP | *tetB* | *gtxA, flfA* | S |  |
| Laying hens | LH-9 * | 2017 | 2.177 | G. anatis DSM16844T | PEN FOT ERY TYLT CLI ENRO NAL CIP SDM STZ SXT ^F^ | n. p. | *gtxA, flfA* | W |  |
| (*n* = 34) | LH-10 | 2017 | 2.162 | G. anatis DSM16844T | PEN FOT ERY TYLT CLI ENRO NAL CIP SDM STZ SXT ^F^ | ND | ND | W |  |
|  | LH-12 * | 2017 | 2.251 | G. anatis DSM16844T | PEN FOT ERY CLI ENRO NAL CIP SDM STZ SMX ^G^ | n. p. | *gtxA, flfA* | S |  |
|  | LH-13 | 2017 | 2.23 | G. anatis GD45 GDD | PEN FOT ERY CLI ENRO NAL CIP SDM STZ SMX ^G^ | ND | ND | S |  |
|  | LH-14 * | 2017 | 2.296 | G. anatis GD45 GDD | PEN FOT ERY TYLT CLI ENRO NAL CIP SDM STZ ^D^ | *tetB* | *gtxA, flfA* | M |  |
|  | LH-17 * | 2018 | 2.115 | G. anatis DSM16844T | PEN FOT ERY TYLT CLI ENRO CIP SDM STZ | n. p. | *gtxA, flfA* | W |  |
|  | LH-16 | 2018 | 2.182 | G. anatis DSM16844T | PEN FOT NEO ERY CLI ENRO NAL CIP SDM STZ SXT TMP | ND | ND | M |  |
|  | LH-30 * | 2019 | 2.078 | G. anatis GD45 GDD | PEN AMP FOT NEO SPE ERY TYLT CLI ENRO NAL CIP SDM STZ SXT SMX TMP | n. p. | *gtxA, flfA* | M |  |
|  | LH-31 | 2019 | 2.191 | G. anatis GD45 GDD | PEN AMP FOT ERY TYLT CLI ENRO NAL CIP STZ SXT SMX TMP | ND | ND | M |  |
|  | LH-19 * | 2018 | 2.332 | G. anatis DSM16844T | PEN FOT OXY TET TGC ERY CLI ENRO NAL CIP SDM STZ SXT TMP | *tetB* | *gtxA, flfA* | S |  |
|  | LH-24 | 2019 | 2.161 | G. anatis GD45 GDD | PEN AMP FOT SPE OXY TET TGC ERY TYLT CLI ENRO NAL CIP SDM STZ SMX TMP | ND | ND | S |  |
|  | LH-25 | 2019 | 2.234 | G. anatis GD45 GDD | PEN AMP FOT NEO SPE OXY TET TGC ERY TYLT CLI ENRO NAL CIP SDM STZ SMX TMP ^B^ | ND | ND | S |  |
|  | LH-45 | 2020 | 2.167 | G. anatis GD45 GDD | PEN AMP FOT NEO SPE OXY TET ERY TYLT CLI ENRO NAL CIP SDM STZ SXT SMX TMP ^C^ | ND | ND | S |  |
|  | LH-26 | 2019 | 2.242 | G. anatis GD45 GDD | PEN AMP FOT NEO SPE OXY TET TGC ERY TYLT CLI ENRO NAL CIP SDM STZ SXT SMX TMP ^E^ | ND | ND | M |  |
|  | LH-27 * | 2019 | 2.41 | G. anatis GD45 GDD | PEN AMP FOT NEO ERY TYLT CLI ENRO NAL CIP SDM STZ SXT SMX TMP | n. p. | *gtxA, flfA* | M |  |
|  | LH-3 * | 2016 | 2.126 | G. anatis DSM16844T | PEN AMOX AMP FOT SPE OXY TET ERY TYLT CLI ENRO CIP SDM STZ SXT TMP | *blaTEM-1, tetB* | *gtxA, flfA* | W |  |
|  | LH-18 | 2018 | 2.225 | G. anatis DSM16844T | PEN FOT OXY TET ERY CLI ENRO NAL CIP SDM STZ SXT SMX TMP | ND | ND | S |  |
|  | LH-23 * | 2019 | 2.145 | G. anatis DSM16844T | PEN AMP FOT NEO SPE OXY TET TGC ERY TYLT CLI ENRO NAL CIP SDM STZ SXT SMX TMP ^E^ | *tetB* | *gtxA, flfA* | S |  |
|  | LH-29 | 2019 | 2.232 | G. anatis GD45 GDD | PEN AMP FOT NEO SPE OXY TET ERY TYLT CLI ENRO NAL CIP SDM STZ SXT SMX TMP ^C^ | ND | ND | M |  |
|  | LH-2 * | 2016 | 2.043 | G. anatis DSM16844T | PEN FOT ERY TYLT CLI ENRO NAL CIP SDM STZ ^D^ | n. p. | *gtxA, flfA* | M |  |
|  | LH-58 | 2020 | 2.341 | G. anatis GD45 GDD | PEN FOT NEO ERY TYLT CLI ENRO NAL CIP SDM STZ SXT TMP | ND | ND | M |  |
|  | LH-59 * | 2020 | 2.232 | G. anatis GD45 GDD | PEN AMP FOT NEO OXY TET ERY TYLT CLI ENRO NAL CIP SDM STZ SMX | *tetB* | *gtxA, flfA* | S |  |
|  | LH-60 | 2020 | 2.227 | G. anatis GD45 GDD | PEN FOT OXY TET ERY TYLT CLI ENRO NAL CIP SDM STZ ^I^ | ND | ND | M |  |
|  | LH-61 * | 2020 | 2.252 | G. anatis GD45 GDD | PEN FOT OXY TET ERY TYLT CLI ENRO NAL CIP SDM STZ ^I^ | *tetB* | *gtxA, flfA* | S |  |
|  | LH-42 | 2019 | 2.323 | G. anatis DSM16844T | PEN AMP FOT NEO SPE ERY TYLT CLI ENRO NAL CIP SDM STZ SXT TMP | ND | ND | S |  |
|  | LH-40 * | 2019 | 2.297 | G. anatis GD45 GDD | PEN AMP FOT NEO SPE OXY TET TGC ERY TYLT CLI ENRO NAL CIP SDM STZ | n. p. | *gtxA, flfA* | S |  |
|  | LH-41 * | 2019 | 2.059 | G. anatis GD45 GDD | PEN AMOX AMP FOT NEO SPE OXY TET ERY TYLT CLI ENRO NAL CIP SDM STZ SXT SMX TMP | n. p. | *gtxA, flfA* | M |  |
|  | LH-48 | 2020 | 2.244 | G. anatis GD45 GDD | PEN FOT NEO SPE ERY TYLT CLI ENRO NAL CIP SDM STZ TMP | ND | ND | S |  |
|  | LH-49 | 2020 | 2.378 | G. anatis GD45 GDD | PEN AMOX AMP FOT NEO SPE OXY TET ERY TYLT CLI ENRO NAL CIP SDM STZ SXT TMP ^J^ | ND | ND | W |  |
|  | LH-57 * | 2020 | 2.371 | G. anatis DSM16844T | PEN AMP FOT NEO OXY TET ERY TYLT CLI ENRO NAL CIP SDM STZ SMX TMP CHL | *aph(3)-IIIa, tetB* | *gtxA, flfA* | M |  |
|  | LH-56 | 2020 | 2.255 | G. anatis GD45 GDD | PEN AMOX AMP FOT NEO SPE OXY TET ERY TYLT CLI ENRO NAL CIP SDM STZ SMX ^K^ | ND | ND | M |  |
|  | LH-46 | 2020 | 2.371 | G. anatis DSM16844T | PEN AMP FOT NEO SPE OXY TET ERY TYLT CLI ENRO NAL CIP SDM STZ SXT SMX TMP ^C^ | ND | ND | M |  |
|  | LH-47 * | 2020 | 2.343 | G. anatis GD45 GDD | PEN AMOX AMP FOT NEO SPE OXY TET TGC AZI ERY TYLT CLI ENRO NAL CIP SDM STZ SXT SMX TMP ^H^ | *blaROB-11, tetB, tetM* | *gtxA, flfA* | W |  |
|  | LH-7 | 2017 | 2.191 | G. anatis GD45 GDD | PEN FOT OXY TET TGC AZI ERY TYLT CLI ENRO CIP SDM STZ SXT SMX TMP | ND | ND | S |  |
| Breeding hens | BH-1 * | 2015 | 2.086 | G. anatis DSM16844T | PEN FOT OXY TET ERY TYLT CLI ENRO CIP SDM STZ | *tetB* | *gtxA, flfA* | M |  |
| (n=17) | BH-8 * | 2017 | 2.279 | G. anatis DSM16844T | PEN FOT SPE OXY TET ERY TYLT CLI ENRO CIP SDM STZ SXT SMX TMP | *floR, tetB* | *gtxA, flfA* | S |  |
|  | BH-22 * | 2019 | 2.201 | G. anatis GD45 GDD | PEN AMOX AMP FOT NEO SPE OXY TET TGC AZI ERY TYLT CLI ENRO NAL CIP SDM STZ SXT SMX TMP ^H^ | *blaROB-11, tetB* | *gtxA, flfA* | S |  |
|  | BH-34 | 2019 | 2.342 | G. anatis GD45 GDD | PEN AMOX AMP FOT NEO SPE OXY TET TGC ERY TYLT CLI ENRO NAL CIP SDM STZ SXT | ND | ND | W |  |
|  | BH-35 * | 2019 | 2.273 | G. anatis GD45 GDD | PEN AMOX AMP FOT NEO OXY TET TGC ERY TYLT CLI ENRO NAL CIP SDM STZ | *blaROB-11, tetB* | *gtxA, flfA* | W |  |
|  | BH-5 * | 2016 | 2.221 | G. anatis GD45 GDD | PEN FOT OXY TET AZI ERY TYLT CLI ENRO NAL CIP SDM STZ SXT TMP | *floR, tetB* | *gtxA, flfA* | S |  |
|  | BH-50 * | 2020 | 2.184 | G. anatis DSM16844T | PEN AMOX AMP FOT NEO SPE OXY TET TGC ERY TYLT CLI ENRO NAL CIP SDM STZ SXT SMX TMP | *blaROB-11, tetB* | *gtxA, flfA* | S |  |
|  | BH-20 * | 2018 | 2.096 | G. anatis GD45 GDD | PEN AMOX AMP FOT OXY TET ERY CLI ENRO CIP SDM STZ SXT | *blaTEM-1, tetB* | *gtxA, flfA* | M |  |
|  | BH-21 | 2018 | 2.274 | G. anatis DSM16844T | PEN AMOX AMP FOT OXY TET ERY CLI ENRO NAL CIP SDM STZ SXT SMX TMP | ND | ND | M |  |
|  | BH-55 | 2020 | 2.32 | G. anatis GD45 GDD | PEN AMOX AMP FOT NEO SPE OXY TET ERY TYLT CLI ENRO NAL CIP SDM STZ SXT TMP ^J^ | ND | ND | M |  |
|  | BH-11 | 2017 | 2.326 | G. anatis GD45 GDD | PEN FOT ERY TYLT CLI ENRO CIP SDM STZ SXT TMP | ND | ND | S |  |
|  | BH-36 * | 2019 | 2.248 | G. anatis GD45 GDD | PEN FOT GEN NEO SPE ERY TYLT CLI ENRO NAL CIP | n. p. | *gtxA, flfA* | M |  |
|  | BH-44 * | 2020 | 2.304 | G. anatis GD45 GDD | PEN AMP FOT NEO SPE OXY TET TGC ERY TYLT CLI ENRO NAL CIP SDM STZ SXT SMX TMP ^E^ | *tetB* | *gtxA, flfA* | M |  |
|  | BH-51 * | 2020 | 2.317 | G. anatis GD45 GDD | PEN AMOX AMP FOT NEO SPE OXY TET ERY TYLT CLI ENRO NAL CIP SDM STZ SMX ^K^ | *blaTEM-1, tetB* | *gtxA, flfA* | S |  |
|  | BH-52 | 2020 | 2.39 | G. anatis GD45 GDD | PEN AMOX AMP FOT SPE OXY TET ERY TYLT CLI ENRO NAL CIP SDM STZ SMX TMP ^A^ | ND | ND | S |  |
|  | BH-53 * | 2020 | 2.33 | G. anatis GD45 GDD | PEN AMOX AMP FOT NEO SPE OXY TET ERY TYLT CLI ENRO NAL CIP SDM STZ SMX ^K^ | *blaTEM-1, dfrK, tetL* | *gtxA, flfA* | S |  |
|  | BH-32 * | 2019 | 2.236 | G. anatis DSM16844T | PEN FOT OXY TET TGC ERY CLI NAL CIP SDM | *tetB* | *gtxA, flfA* | M |  |
| Ornamental hen (n=1) | OH-43 * | 2019 | 2.143 | G. anatis DSM16844T | PEN AMP FOT NEO SPE ERY TYLT CLI ENRO NAL CIP SDM STZ | n. p. | *gtxA, flfA* | M |  |

* strains selected for whole genome sequencing;

^A-K^ strains with the same AMR profile

ND – no data; n. p. – no presence; Biofilm formation; S - strong biofilm producer, M - moderate biofilm producer, W - weak biofilm producer
